# Partial migration in savanna elephant populations distributed across southern Africa

**DOI:** 10.1038/s41598-018-29724-9

**Published:** 2018-07-27

**Authors:** Andrew Purdon, Michael A. Mole, Michael J. Chase, Rudi J. van Aarde

**Affiliations:** 1 Conservation Ecology Research Unit, Department of Zoology and Entomology, University of Pretoria, Pretoria, 0028 South Africa; 2 Elephants Without Borders, Kasane, Botswana

## Abstract

Migration is an important, but threatened ecological process. Conserving migration requires the maintenance of functional connectivity across sufficiently large areas. Therefore, we need to know if, where and why species migrate. Elephants are highly mobile and can travel long distances but we do not know if they migrate. Here, we analysed the movement trajectories of 139 savanna elephants (*Loxodonta africana*) within eight clusters of protected areas across southern Africa to determine if elephants migrate, and if so, where, how and why they migrate. Only 25 of these elephants migrated. Elephants are a facultative partially migratory species, where only some individuals in a population migrate opportunistically, and not every year. Elephants migrated between distinct seasonal ranges corresponding to southern Africa’s dry and wet seasons. The timing of wet season migrations was associated with the onset of rainfall and the subsequent greening up of forage. Conversely, the duration, distance, and the timing of dry season migrations varied idiosyncratically. The drivers of elephant migration are likely a complex interaction between individual traits, density, and the distribution and availability of resources. Despite most migrations crossing administrative boundaries, conservation networks provided functional space for elephants to migrate.

## Introduction

Migration is an ecologically important process that can have consequences for individual fitness, population demography^1–3^, and the structure and dynamics of ecosystems^4,5^. However, migration is increasingly threatened by anthropogenic pressures, habitat fragmentation, and climate change^1,6,7^. Identifying migratory species and the ultimate and proximate drivers of their migratory tendencies is therefore of conservation importance. The savanna elephant (*Loxodonta africana*) is one of Africa’s most iconic and well-studied large mammal species. Yet, whether elephants migrate or not remains unsubstantiated in the scientific literature due to a lack of empirical evidence, small sample sizes, and inadequate analytical routines. Indeed, whether elephants do migrate, and if so, where, how and why they migrate needs to be investigated.

Migration, defined here as a repeated seasonal movement between two non-overlapping regions^8^, is an adaptive response to living in seasonal environments and to the spatiotemporal distribution of resources^8^. For large herbivorous mammals, migration is commonly linked to the seasonal distribution of resources related to forage availability and quality^7,9–11^. Additional reasons for migration may include predator avoidance^12^, intra-specific competition^11^ and parasite avoidance^13,14^. However, migration is multifaceted and there are a number of different types of migrations that take place at various scales^8^.

At the population scale, migration can be considered as complete, where all individuals migrate, or partial, where only some individuals in a population migrate^15^. Partial migration seems to be the norm amongst large mammals^16^. At the individual scale, migration can be obligate, where individuals migrate annually, or facultative, where individuals do not migrate annually but rather opportunistically in response to local environmental conditions^8,10^. Facultative migration might be more common than obligate migration in some taxa^10^, but supporting long-term studies are scarce.

Unravelling the drivers of migratory behaviour or tendencies can be complex as they often involve interactions between intrinsic, environmental, and density dependent factors^15,17^. For example, whether or not populations or individuals migrate may depend on the seasonality of the environment they live in. Large mammals that live in seasonal environments where resources vary spatiotemporally are more likely to migrate than those living in less seasonal environments^10,18,19^. If seasonal changes are predictable, then migration may evolve to be obligate, but in seasonal environments that are less predictable, facultative migration is expected^20^. Partial migration may also be density dependent^15,21^. To avoid intra-specific competition, some populations with high densities are more likely to have migratory individuals than others^11,19^. Density can also affect an individual’s tendency to switch between migratory and non-migratory movements^17^. Lastly, phenotypic characteristics, such as body size, sex, or age may influence an individual’s competitive ability or ability to avoid predation^15^, which in turn may alter the propensity of an individual to migrate^15,17^.

**Figure 1:**
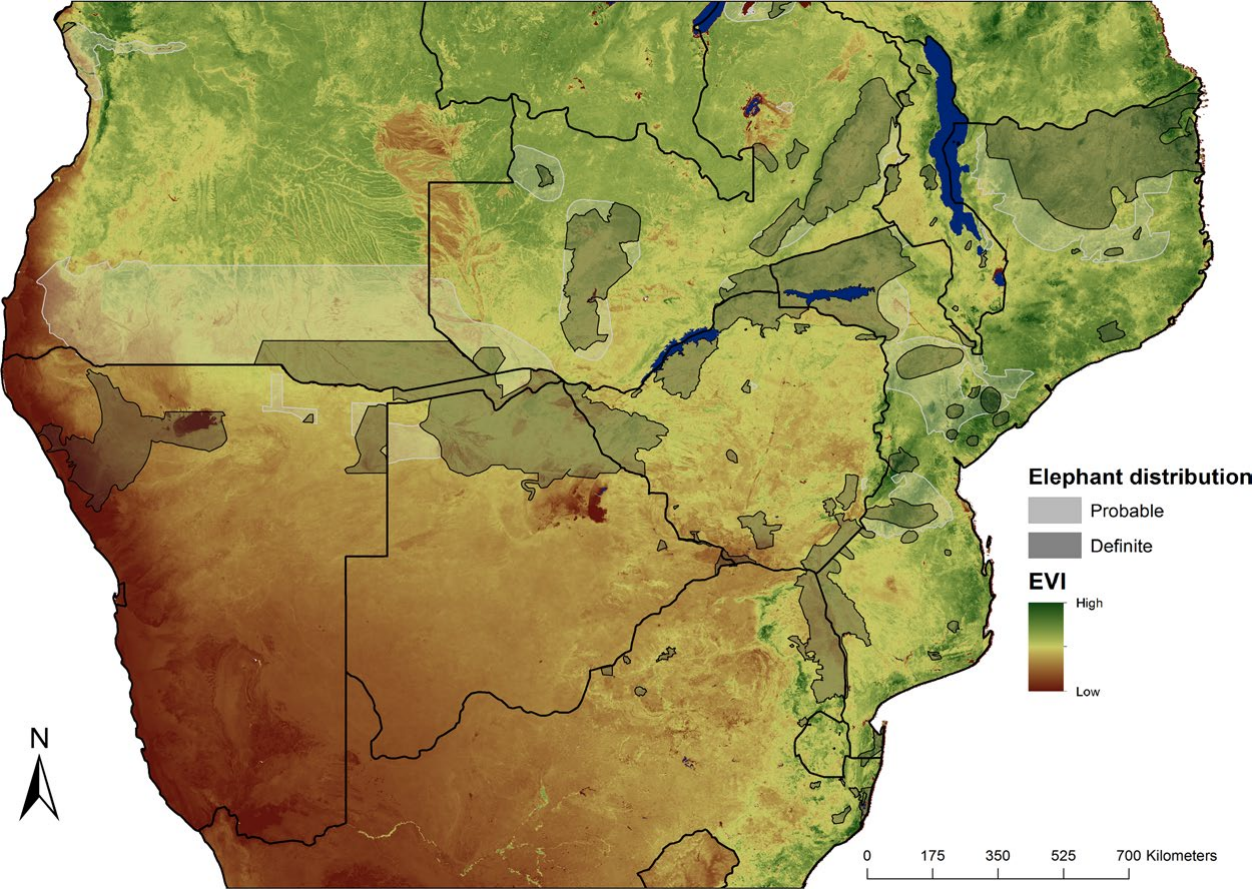
Map of southern Africa illustrating the known and probable present-day savanna elephant distribution^58^. The map colour represents a measure of primary productivity (the mean Enhanced Vegetation Index (EVI) over a 16-year time period from 2000 to 2016; see Supplementary Information Materials and Methods for details). This map was generated with the software ArcGIS ver. 10.3.1 (https://www.arcgis.com/features/index.html).

Savanna elephants are widely distributed and commonly occur where rainfall and primary productivity vary seasonally (Fig. 1). Across their distributional range, other co-occurring large mammal species such as Burchell’s zebra (*Equus burchellii*)^7,11^ and blue wildebeest (*Connochaetes taurinus*)^7^ migrate in response to seasonal environments. Therefore, like other co-occurring large mammals, elephants should exhibit a range of movement tactics which includes migration^8,10,18^. Early accounts of possible migratory behaviour in elephants stem from several count and telemetry studies documenting long distance movements in Namibia and East Africa^22–26^. Recent spatially explicit studies suggest spatial separation of seasonal ranges^27–32^. In these studies, migration seems to be induced by seasonal rainfall and the speculated changes in forage availability. However, these studies involved only a few individuals in a small part of the distributional range of savanna elephants. These studies also failed to give a clear definition of what type of migration they deduced. We therefore do not know if elephants are partially or fully migratory, if migration is facultative or obligatory, or what drives migration.

**Figure 2:**
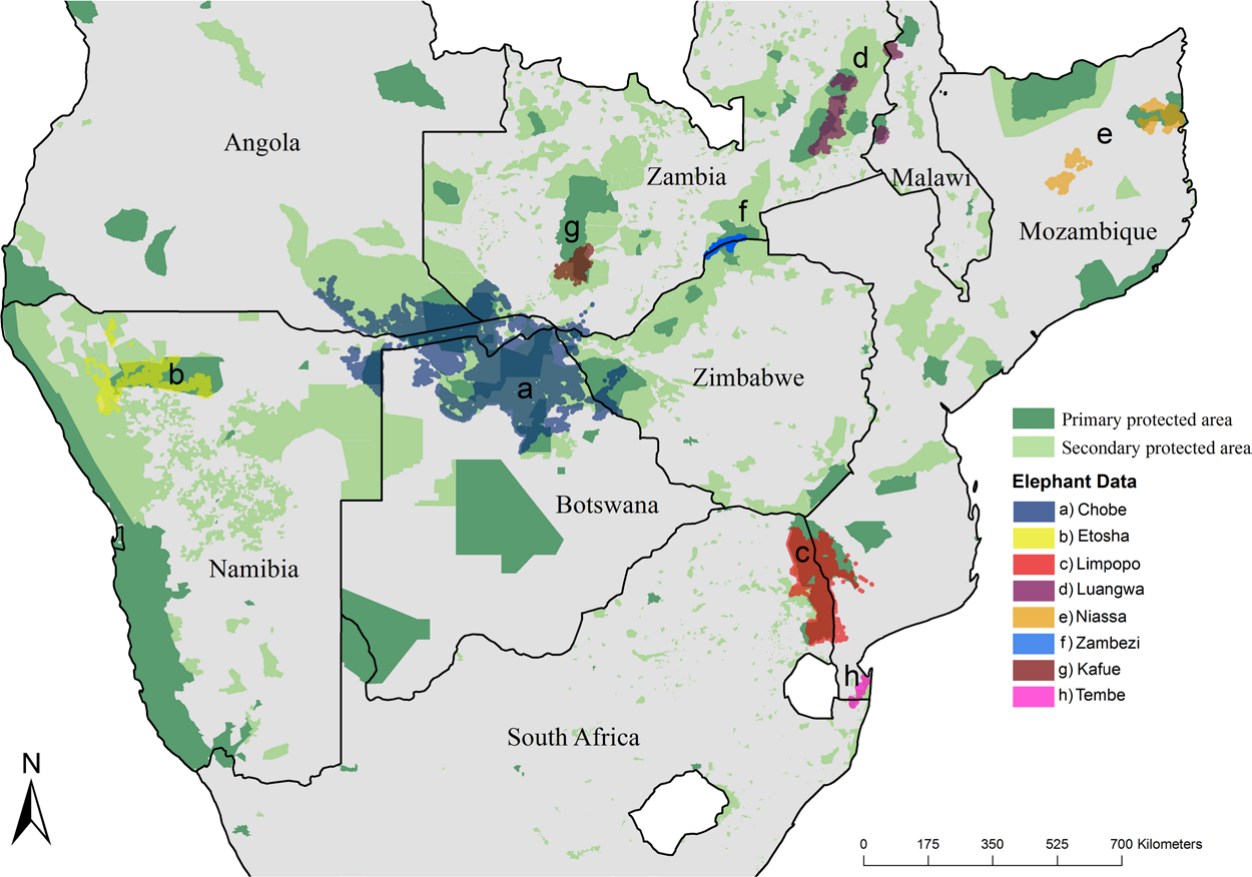
Map of southern Africa showing the distribution of the elephant location data. The elephant location data are coloured according to cluster. Protected areas were sourced from the World Database on Protected Areas^52^. Primary protected areas are national parks and game reserves and secondary protected areas are game/wildlife management areas, communal conservancies, hunting reserves, and forest reserves. This map was generated with the software ArcGIS ver. 10.3.1 (https://www.arcgis.com/features/index.html).

In the present study, we analysed the movements of 234 savanna elephants within eight clusters of protected areas distributed across southern Africa, to answer a) do elephants migrate? and if so, b) where and why do elephants migrate? The study area comprised of protected areas containing 74% of the continent’s estimated 352,271 elephants^33^ (Fig. 2). Habitats varied from predominantly arid shrublands in Namibia to mesic woodlands in Mozambique (Fig. 1). We defined migration as a movement between two non-overlapping seasonal ranges. Using two independent analytical routines, we classified movements within a year as migratory or not. We then investigated the patterns and drivers of elephant migration. Based on early accounts of possible elephant migration, we predicted that at the population scale elephants are partially migratory. Furthermore, we expected that only some populations would have migratory individuals, specifically those in more seasonal and arid environments. At the individual scale, in migratory populations, we expect that migratory elephants are facultative migrants due to the unpredictability of African savannas^34^. Additionally, we predict that more males would migrate than family herds, because, males are typically larger and their movements are uninhibited by young calves^35^.

Large herbivores typically migrate during periods of plant growth to access high quality forage and then return to avoid adverse weather conditions or limited resources when seasons change^4^. Elephants seek out greener than expected vegetation throughout the year^36^ and their home ranges are limited to permanent water sources in the dry season^37,38^. Therefore, we expect that the timing and duration of migrations would depend on the distribution of forage across a landscape and seasonal rainfall, as previously documented for large African ungulates^11,39^. In line with this expectation, we test the hypothesis that the migration of elephants is driven by the spatiotemporal distribution of resources, in particular food and water. We expect migrations to be associated with the onset of the wet season, when ephemeral water sources fill up and the distribution of surface water no longer limits the distribution of elephants. Furthermore, we expect elephant dry season ranges to be close to permanent water sources and the ranges that they migrate to, to be further away from permanent water sources where ephemeral water sources are likely available^30,38^. We also expect elephants to migrate to areas with higher primary productivity, particularly if water is no longer a limiting factor, for example during the wet season. Lastly, if migration is density dependent, we expect elephants to migrate from areas of high density to areas of low density during the wet season to avoid intra-specific competition.

## Results

The location data from the original sample size of 234 elephants was reduced to 139 elephants after discarding elephants that had insufficient location data (i.e. less than a full year of location data, please see Supplementary Information Fig. S1). Here and throughout this manuscript, we refer to a year as a unit of time to express duration i.e. 12 months. The 139 elephants yielded 234 years of location data (Table 1, Fig. 2). Of the 139 elephants, 72 had a single year worth of location data, while 67 had multiple years of data (41 individuals had two years, 24 had three years, and two had four years of location data). Of the 139 elephants, 97 were adult females and 42 were adult males.

**Table 1:** Summary of the number of elephants and years of location data sampled in the present study as well as the number of years and elephants classified as migratory using both the Net Squared Displacement method and the overlap method^54^ for each cluster.

Cluster	Countries	Sampling period	Number of elephants after filtering			Number of years of locational data after filtering			Number of migrations			Number of migratory elephants			Number of elephants withmultiple migrations			Proportion migratory elephants
			N	males	females	N	males	females	N	males	females	N	males	females	N	males	females	
Chobe	Botswana/Angola/Namibia/Zimbabwe/Zambia	2001–2014	63	27	36	95	37	58	19	7	12	15	6	9	4	1	3	0.24
Etosha	Namibia	2002–2008	10	4	6	12	6	6	3	1	2	3	1	2	—	—	—	0.3
Kafue	Zambia	2003–2005	7	4	3	7	4	3	—	—	—	—	—	—	—	—	—	0
Luangwa	Zambia/Malawi	2004–2010	16	2	14	37	5	32	2	—	2	1	—	1	1	—	1	0.06
Limpopo	South Africa/Mozambique	2002–2016	28	2	26	60	5	55	4	—	4	4	—	4	—	—	—	0.14
Niassa	Mozambique	2007–2011	8	2	6	16	4	12	2	2	—	1	1	—	1	1	—	0.13
Tembe	South Africa/Mozambique	2000–2002	2	1	1	2	1	1	—	—	—	—	—	—	—	—	—	0
Zambezi	Zambia/Zimbabwe	2004–2006	5	—	5	5	—	5	1	—	1	1	—	1	—	—	—	0.2
Total			139	42	97	234	62	172	31	10	21	25	8	17	6	2	4	0.18

The NSD (Net Squared Displacement) method (see Supplementary Information Fig. S2 for details) classified 120 of the 234 years as non-migratory (52%), and 114 as migratory (48%). The overlap method (see Supplementary Information Fig. S2 for details) classified 179 as non-migratory (76%), and 55 years as migratory (24%). A total of 31 years (20%) were classified as migratory in both the NSD and overlap method (Table 1). All results reported on from this point forward are based on the 31 migrations classified by both methods as migratory. The 31 years classified as migratory comprised of 25 individuals: 17 female and eight male elephants. There was no significant difference between the proportion of female and male elephants that were analysed (♂ 97; ♀ 42) in comparison to those that migrated (♂ 17; ♀ 8; Pearson’s Chi-square test, χ² = 1.6 × 10^−30^, P = 1.0).

**Figure 3:**
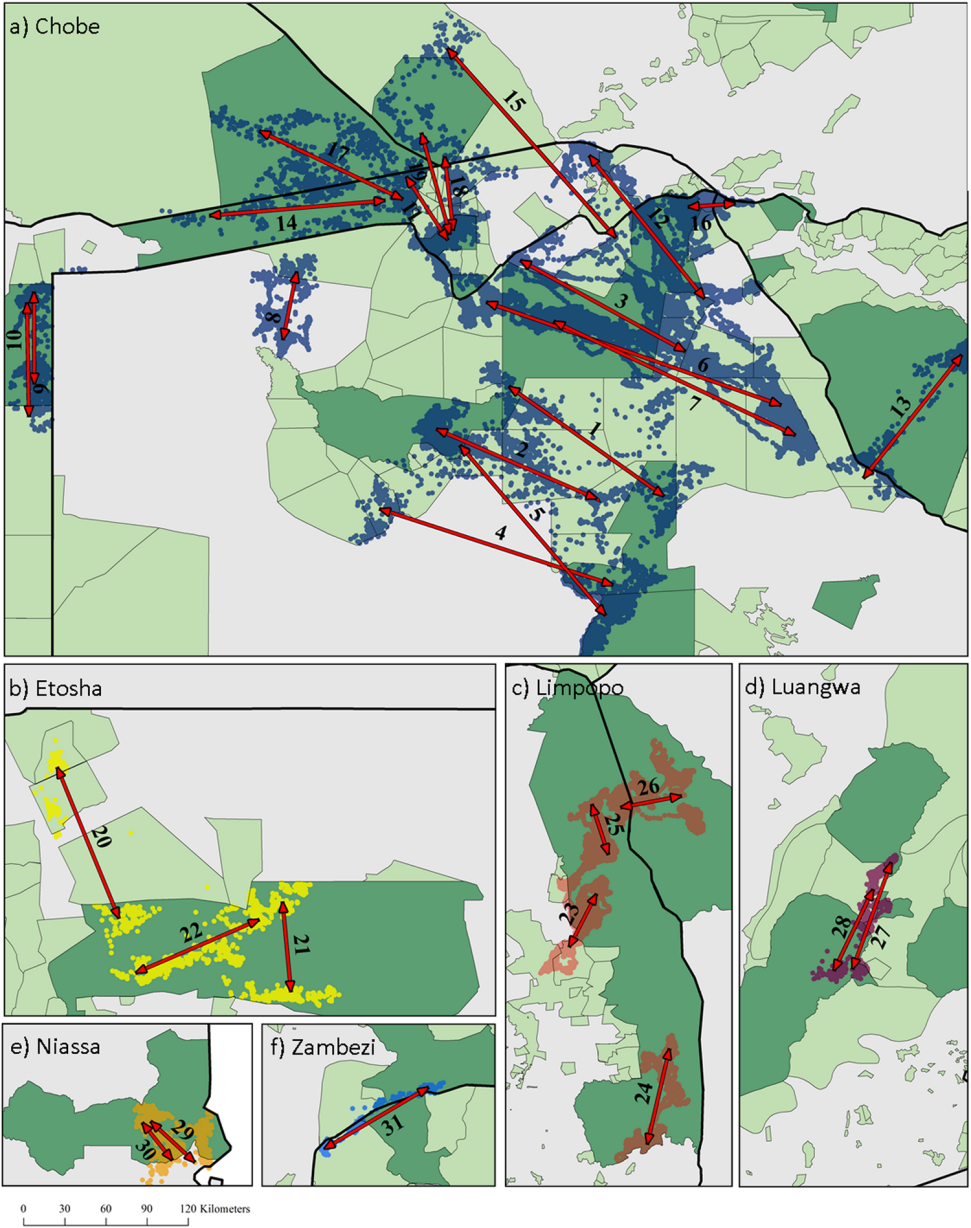
The direction of the 31 classified migrations in each cluster. The arrows only show direction and do not represent the actual migratory route. The numbers correspond to migration ID**’**s in Fig. 4. Details on the characteristics for each migration can be found in Supplementary Information Table S1. These maps were generated with the software ArcGIS ver. 10.3.1 (https://www.arcgis.com/features/index.html).

Migrations occurred in six of the eight protected area clusters and no migrations took place in Kafue or Tembe (Fig. 3). The proportion of individuals that migrated differed between clusters, but remained low (Table 1). Most migrations took place within protected area networks with only seven migrations moving into unprotected areas (Fig. 3, Supplementary Information Table S1). However, 26 migrations out of the 31 extended beyond primary protected area (IUCN categories 1-IV) boundaries but not beyond those of secondary protected areas (IUCN categories V and VI) (Fig. 3). Ten migrations crossed international borders (Supplementary Information Table S1).

**Figure 4:**
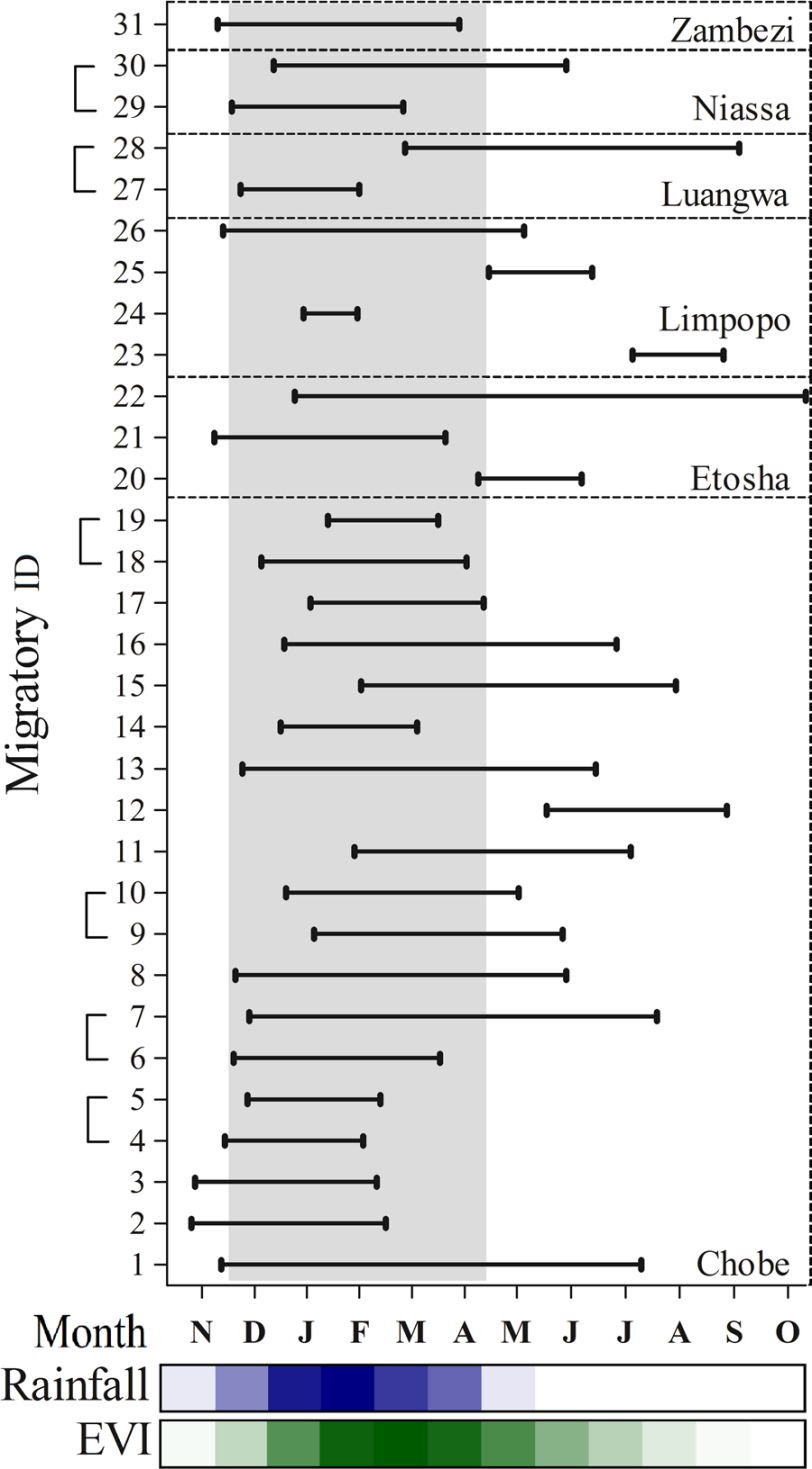
Timing characteristics for each of the 31 classified migratory years. Each line represents the period that the elephant was in its away seasonal range and illustrates the leave day and return day of the migrations. The rainfall and EVI gradient bars (light to dark) represent mean monthly rainfall and EVI values for the entire study area from 2000–2016 (see Supplementary Information Materials and Methods for details). The shaded vertical bar represents southern Africa**’**s wet season. Brackets highlight annual migrations undertaken by the same individual. The numbers correspond to migration ID**’**s in Fig. 3 and Supplementary Information Table S1.

Most of the migratory elephants with more than one year of location data switched between being migratory and non-migratory. Of the 25 elephants that migrated, 15 had multiple years of location data (seven elephants had two, seven had three years, and one had four years of data). Nine of these 15 individuals switched between being non-migratory and migratory between years, four migrated every year, and two migrated twice but not during consecutive years (Table 1, Supplementary Information Table S1). For elephants that migrated more than once, the migratory route, distance and timing were similar. However, the migratory timing differed between years for an individual (migration ID 27 and 28) that migrated twice in the Luangwa cluster (Fig. 4). For elephants that switched between being migratory and non-migratory, non-migratory movements appeared idiosyncratic with no clear pattern between switching individuals.

**Figure 5:**
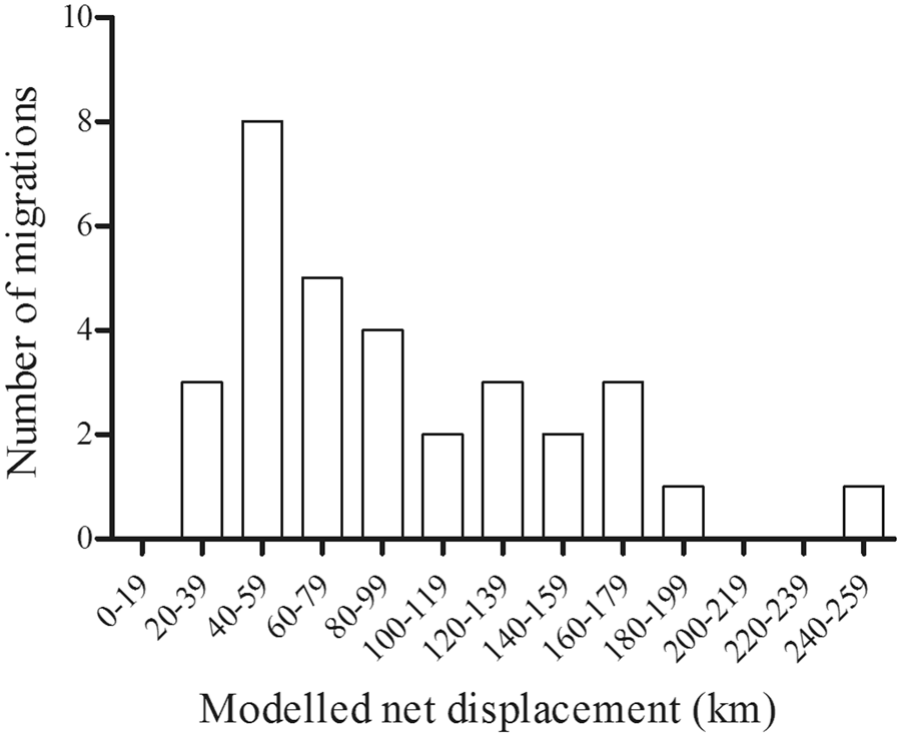
Frequency histogram of one-way migratory modelled net displacements for the 31 classified migrations.

Migratory characteristics varied between individuals (Figs 4, 5, Supplementary Information Table S1). One-way migration distances ranged from 20 to 249 km (Fig. 5) with no clear pattern between sex or cluster. The longest migration took place in Etosha (migration ID 22) while the shortest migration took place in Chobe (Fig. 3: migration ID 16). In total, 77% of all departures took place during November and January (Fig. 4). This pattern correlated with the onset of the wet season and the subsequent greening up of vegetation (Fig. 4). The duration of time elephants spent in their away migratory ranges also corresponded with the wet season for most migrations, except in six (migration ID 12, 20, 22, 23, 25, 28; Fig. 4). While departure dates were consistent, the dates elephants migrated back to their dry season ranges varied greatly but most returned before the end of the dry season (Fig. 4).

The selection of wet season ranges by migratory elephants could not be explained by differences in EVI, distance to water or elephant density (see Supplementary Information Table S3a). The model that best explained the selection of wet season ranges was the null model with an AICc weight of 0.32 (see Supplementary Information Table S3a). AICc differences across the candidate models were relatively low. Furthermore, none of the relationships were significant (primary productivity (P = 0.95); distance to water (P = 0.71); elephant density (P = 0.62), see Supplementary Information Table S3b).

## Discussion

We set out to investigate if elephants migrate, and if so where, how and why they migrate? Our assessment illustrates that only some savanna elephants do migrate, but that migrations take place in most regions where elephants are distributed and most migrations extend beyond the boundaries of primary protected areas. Elephants should be considered partial and facultative migrators that may migrate in response to seasonal rainfall. However, EVI, water availability, and population densities did not explain our recorded migratory patterns.

Migration is not a simple process and unravelling the migratory tendencies of a species requires investigating patterns at the individual and population scale. By analysing the yearly movement data of 139 savanna elephants from eight clusters of protected areas across southern Africa, we determined that like several other large mammals^16^, the elephant is a partially migratory species. In other words, only some individuals in a population migrate. Indeed, based on our strict analytical routines, where two prescribed methods were used to classify migrations, we found that overall very few elephants migrated (18%). This was also the case within each of the populations, albeit slight differences in the proportion of migratory elephants. For example, Etosha, a highly seasonal and arid environment, had the most migratory elephants (30%), as opposed to the less seasonal but more mesic Niassa (13%). At the population scale, migration is generally more common in seasonal and predictable environments^18,19^. Therefore, we expected this to be the case for elephants across their distributional range. These patterns may exist, but we could not verify this because so few individuals migrated. However, it is important to note that despite large inter-population differences in environmental conditions and elephant densities, most of the protected area clusters harboured migratory individuals.

Amongst partially migratory species, individuals can be obligate or facultative migrants^8^. Facultative migration, where individuals do not migrate every year, has been documented in several ungulate species^10,17^. Facultative migration is viewed as an adaptive response to living in variable seasonal environments and is as an opportunistic response driven by proximate local conditions^10,20^. Of the 67 elephants we monitored for more than one year, 11 switched between being migratory or non-migratory. This relatively low proportion is comparable to one of the only long-term studies to have addressed switching behaviour in another large mammal, the elk (*Cervus elaphus*), where only 16% of elk switched between migratory and resident^17^. Switching suggests that elephant migration is facultative and not a fixed obligate response. It also highlights a high degree of behavioural flexibility in elephants. African migratory mammals often rely on environmental cues such as rainfall to begin their wet season migration^39^. However, in highly variable environments, environmental cues at one’s present location may be unreliable predictors for distant locations. These cues may also vary between years. Therefore, movement behaviour needs to be flexible enough to cope with this uncertainty. To this end, elephants show a high degree of movement plasticity and likely only migrate opportunistically if conditions at a point in time are conducive for migration.

In our study, most of the migratory elephants (77%) migrated at the onset of the wet season, migrating towards a wet season range. For these elephants, their annual migration consisted of two distinct seasonal long-distance movements that were directional, occurring over a short period and taking place between two non-overlapping wet and dry season ranges, as have been documented by others^27,30^. Other migratory characteristics however were highly variable and idiosyncratic. This includes the time spent in their wet season ranges and the timing of return migrations towards their dry season ranges. Migrations also varied in distance and between clusters with inconsistencies between individuals and years. The elephant movement patterns that were classified as migratory and that took place within the dry and wet seasons support our conclusion that elephants do migrate seasonally. However, for the elephants that were classified as migratory but that did not migrate within the expected seasonal windows, nomadic or exploratory movements, where individuals undertake a round-trip or return journey, cannot be ruled out. To unravel these differences in movements, long term movement data over continuous years is required.

Theory suggests that benefits for migratory individuals include exploiting changes in forage abundance or quality, accessing spatiotemporally limited resources^4^, escaping competition^19^, avoiding predation^12^ or parasite pressure^14^. For elephants, predation and parasite pressure are likely not factors that influence migration and we could not address them in the present study. However, we could address the spatiotemporal distribution of resources, as well as the competition that may arise in competing for those resources. Elephants tend to congregate around permanent water sources during the dry season^37,38,40^, leading to a local increase in elephant density. We hypothesised that at the onset of rain, elephants migrate to areas further away from permanent water sources where they can avoid competition and access new growth forage higher in primary productivity than what they would have experienced if they had stayed within their dry season ranges. However, while our results do not support this hypothesis, for reasons listed below we cannot reject it.

Firstly, migratory herbivores generally track seasonal changes in food quality rather than food abundance^4^. This is apparent in several African ungulates that tend to select more nutritious open grasslands during the wet season^11^. Our measure of primary productivity (EVI) is more indicative of food abundance rather than food quality, and certainly is unable to detect the nutritional differences in vegetation types^41^. The consistent timing of wet season migrations at the onset of rainfall as well as the apparent areas that elephants are migrating to, suggests that elephants migrate to access new growth forage in wet season ranges where food quality is higher^41^. For example, during the wet season in northern Botswana, elephants migrated to the Makgadikgadi and Nxai Pan National Parks (see Fig. 3: migration ID’s 1, 2, 4, and 5), areas dominated by grasses, which typically have higher protein and mineral content during the wet season^42^. Secondly, migratory species often cannot remain in the areas they have migrated to due to limiting or constraining factors. For example, in temperate regions, snowfall often limits foraging ability and forage availability, forcing animals to migrate towards warmer climates or lower elevations^10,20^. In African savannas, water is considered to be the most limiting factor constraining seasonal habitat use in migratory species^4^. The availability of surface water within migratory wet season ranges is likely the limiting factor driving return migrations in elephants to dry season ranges where permanent water sources are available. In this regard, inter-annual variations in the amount of rainfall may explain the idiosyncratic patterns observed in the duration of time spent in wet season ranges and the highly inconsistent timing of return migrations. Our results show that elephants were not selecting areas further from permanent water sources during the wet season. However, we could not quantify the amount (volume or area) of permanent water available within their ranges. For instance, a small permanent water hole would affect the Euclidean distance to permanent water even though it may only be able to supply a few elephants with water. We also cannot rule out inter-population differences. For example, in the Luangwa, migrations took place in a north-south direction along the Luangwa River (Fig. 3: migration ID 27 and 28). Similarly, in Kruger National Park, the distribution of artificial water points means that elephants are almost always within close proximity to permanent water^43^. A more detailed analysis within each population could provide better insight.

Lastly, theory suggests that density dependence is an important driver of partial migration^15,21^. Migration is viewed as a tactic to avoid intra-specific competition and populations with high densities are often more likely to have migratory individuals^10^. This has been demonstrated in a number of large herbivores, including roe deer (*Capreolus capreolus*)^10,19^, elk (*Cervus elaphus*)^17^, zebra (*Equus burchelli*) and blue wildebeest (*Connochaetes taurinus*)^11^. Our results suggest that elephants do not migrate towards areas with fewer elephants. However, intra-specific competition is not only dependent on density but also on the availability of resources within a range. Density on its own is therefore not an accurate measure of possible intra-specific competition. We lacked sample size and density data at the right scales to thoroughly assess density dependent competition and its possible effect on migratory tendencies in elephants. Future studies should investigate density dependence at the population scale, with density measurements across the entire population and at the individual scale, with local density estimates.

While trying to unravel why elephants migrate, we also need to assess why only some elephants migrate and others do not. Sex, age, body size and even personality may influence an individual’s migratory tendencies^10,15,17,44,45^. In the present study, there were no differences in migration tendencies between sexes, and because all females collared in the present study were part of a breeding herd that typically consists of individuals of various ages, it is unlikely that age played a role. The reasons may come down to a combination of environmental variables and an individual’s characteristics or phenotypic traits. These traits may include body size that in turn may influence an individual’s energy demands, dominance, or competitive ability^45,46^. However, we also cannot rule out the possibility that elephant migration may be inherent, stemming from certain genetic traits.

Although our study focused on identifying and classifying migratory behaviour, one must recognise that elephants are very mobile and their movements can be highly variable. Elephants may employ a large continuum of movement behaviour that not only includes migration but may also include highly variable home range or resident behaviour^40^. Furthermore, environmental conditions conducive for migration, for example, broad landscape variability in resources, may also drive nomadic behaviour^18^. Particularly in less seasonal and unpredictable environments^18^. In several cases where migration was not identified, nomadic movements may have taken place. Investigating these movement types and other long-distance movements could be important for understanding the ecology and conservation of elephants. Our study represents the first attempt at identifying and investigating migration in savanna elephants across multiple populations. While our study is not complete, it is certainly a step in the right direction and forms a baseline for future studies. Primarily we wanted to identify with certainty whether elephants migrate. As such, we used two independent methods to classify migration. We acknowledge that this analytical routine was strict and that there was a large discrepancy between the two methods and less defined migrations may have been overlooked. As such, we were left with very few migratory elephants to analyse. Whether this was a true reflection of reality or a reflection of our method remains to be seen. Nonetheless, we then attempted to unravel fine scale individual patterns of migration from a sample of 25 animals, with data staggered over a 15-year period and across multiple populations. Moving forward, we suggest that the method should be refined and case studies are conducted within each population where individuals are tracked for multiple successive years.

The greatest challenge in conserving migration is maintaining functional connectivity across sufficiently large areas^1,6,47^. Migration is flexible in elephants; however, elephants can only migrate if protected areas have enough functional space for them to do so. Conservation networks consisting of a mosaic of primary and secondary protected areas are being developed to help link isolated populations and enlarge protected areas^47–49^. It is promising to note that while 26 migrations extended beyond primary protected area boundaries, only seven of the elephants entered unprotected areas. In many cases, secondary protected areas between national parks were utilised as corridors by elephants^50^ between their migratory ranges (e.g. between Chobe and Nxai Pan (migration ID 1); Makgadikgadi and Moremi (migration ID’s 2, 4, and 5); and North and South Luangwa (migration ID 27 and 28). The establishment of the Great Limpopo Transfrontier Park around Kruger National Park has also allowed elephants to utilise seasonal ranges beyond the national park boundaries into areas that were previously inaccessible due to fencing. Despite most migrations crossing administrative boundaries, conservation networks provided functional space for elephants to migrate, highlighting the success of conservation initiatives that are striving to maintain and increase connectivity between protected areas.

## Materials and Methods

### Study area

The study area comprised of protected areas containing 74% of the continent’s estimated 352,271 elephants^33^ (Fig. 2). Here we identified eight geographical clusters of protected areas within which elephant populations were known or suspected to be interconnected^50–52^ (Fig. 2). Protected areas were designated as either primary or secondary based on their designation in the World Database on Protected Areas^53^ (Fig. 2). Primary protected areas are national parks and game reserves. Secondary protected areas include game/wildlife management areas, communal conservancies, hunting reserves, and forest reserves.

Habitats varied from predominantly arid shrublands in Namibia to mesic woodlands in Mozambique (Fig. 1). The terrain was relatively flat across most of the study area, except near the Etendeka Mountains in western Namibia and the Muchinga Mountains in Zambia. Most elephants within this study roamed freely and were not confined by artificial boundaries^51^. However, at the time of the study Etosha National Park, Kruger National Park, and Kaudum were partially fenced and Tembe Elephant Reserve was fully fenced^37^.

### Elephant data set

Elephants (n = 234) were captured and collared with African Wildlife Tracking GPS collars (model SM 2000E; African Wildlife Tracking, Pretoria, South Africa) between December 2002 and December 2014. All individual animals were fitted with GPS collars for at least one year. We, therefore, obtained a time series of GPS locations with a fixed interval between 1 and 24 hours. Subsequently, we resampled GPS data to a single location per day (median location).

### Ethics statement

All aspects of the study were subject to ethical review and were approved by the Animal Ethics Committee of the University of Pretoria (AUCC-040611-013) and were carried out in accordance with international and national guidelines.

### Data availability

The datasets analysed during the current study are available from the corresponding author upon reasonable request. The data are not publicly available due to the sensitivity of locational data of a highly-poached species.

### Classifying migration

Annual migration is a back and forth movement that takes place between two spatially distinct seasonal ranges in one year^8^. Therefore, the sampling unit was a full year of elephant location data and the objective was to classify every year of location data as migratory or non-migratory. Throughout this manuscript, we refer to a year as a unit of time to express duration i.e. 12 months. As suggested by Cagnacci^54^ we used more than one method to classify annual migration. The analytical procedure consisted of three steps: (1) filtering the location data to consist of elephants with full years of data; (2) classifying yearly location data as either migratory or non-migratory; and (3) quantifying and analysing the characteristics of each migration in terms of duration, timing, and distance. The methodological framework is outlined in a flow diagram in Supplementary Information Fig. S1.

#### Filtering the data into full years of location data

The starting date of a year may influence the ability of the methods to identify a migration^54^ e.g. if the starting date begun after the onset of migration, the start of a migration may be missed. Elephants are less mobile during the dry season and more faithful to dry season ranges across years^30,37,40^. They are therefore more likely to migrate during the wet season. Subsequently, for standardization and to maximise sample size, we assigned the starting date for each year as the earliest date in a wet season since the date of collaring. For all clusters the starting date was set as 01 November except for Etosha (01 December) and Zambezi (01 January). Based on the above starting dates, we filtered the elephant (n = 234) location data by discarding elephants that had less than one year of location data and less than 15 locations within a month.

#### Classifying migratory and non-migratory movement

To classify a year of location data as migratory or non-migratory we used two independent methods^54^: (1) overlap of seasonal ranges (overlap^54^), and (2) Net Squared Displacement (NSD^55^). A year of location data was classified as migratory if both the NSD method and the overlap method classified it as migratory.

We used the flexible approach of Cagnacci^54^ that delimits seasons by shifting time windows (resolution of one month) to obtain all possible combinations of three seasonal ranges within a year. For a year to be classified as migratory there must be: (1) a low degree of overlap between the first and second successive range i.e. spatial separation between seasonal ranges, and (2) a high degree of overlap between the first seasonal range and the third seasonal range i.e. the individual returned to the starting seasonal range (see Supplementary Information Fig. S2 for details). We computed the overlap between seasonal ranges using the *kernaloverlaphr* function of the R package adehabitat^56^ (href smoothing factor). The function calculates the Bhattacharyya’s affinity index (BA) index, which quantifies the degree of similarity among probability surface estimates on a scale from zero (no overlap) to one (complete overlap)^57^. We defined a successive season threshold value of BA = 0.15^54^. If the overlap between the first and second successive range was below 0.15 (i.e. no overlap), we further distinguished between migratory and non-migratory by assessing whether the individual returned to a range similar to that of its starting seasonal range. If there was high overlap between the possible return seasons (BA > 0.50), we defined the year as migratory.

Following Bunnefeld^55^, we used the NSD method to classify a year of location data as either migratory or non-migratory. NSD measures the cumulative squared displacement between locations from the starting date of a year over the period of a full year^55^. Using the *nls* function in R, we fitted nonlinear models (corresponding to migratory or non-migratory) to the plotted NSD of each year of location data for each elephant^55^. The best model for each year was selected using AIC_c_ and AIC_c_ weights^17^. If a migratory model was chosen as the best-fit model, the year was classified as migratory (see Supplementary Information Fig. S2 for details). Migratory years with migratory durations fewer than 30 days were discarded to avoid short exploratory movements being classified as migrations. This was consistent with the overlap method, which only considered ranges at a resolution of one month.

#### Quantifying and analysing migration patterns

For all years classified as migratory in both the NSD and overlap methods, we assessed the patterns of migration using the modelled parameters from the migratory NSD model. Patterns of the migrations that we extracted were duration, timing, and distance (see Supplementary Information Fig. S3 for details). The one-way migration distance was calculated as the asymptotic height of the modelled NSD and the leaving and return date were calculated as the day at which the migration reached half its asymptotic height^54^.

### Analysing differences in seasonal ranges of migratory elephants

To determine if elephants that migrated selected ranges that differed from the ranges they migrated from we compared primary productivity, elephant density, and distance from water of the range they migrated from and the range they migrated to (Supplementary Information Table S2). The timing of each seasonal range was classified using the modelled timings from the migratory NSD model (see Supplementary Information Fig. S3). To quantify the seasonal ranges, we calculated the centroid point from the location data that fell between leave days and returns days of each migration. We then buffered the centroid point using a quarter of the migration distance to assess the core area of each range. The buffered areas were assumed to represent core seasonal ranges.

For each of the ranges elephant density, mean Enhanced Vegetation Index (EVI), and the mean distance from permanent water was extracted (*see below*). Elephant density (elephants/km^2^) was calculated as the total number of elephants estimated within a demarcated protected area divided by the area. Data on savanna elephant population estimates came from the African Elephant Database^58^ and our own databases (see^59^ for details). To minimize error within the dataset we only included estimates with a survey reliability of A or B^60^. Elephant counts were not conducted every year. Therefore, we used the count closest to the year the elephant migrated. If count data was unavailable for a certain area, then that data point was excluded from the analysis (Supplementary Information Table S2).

We used the Enhanced Vegetation Index (EVI) as an index of primary productivity^61^. We chose EVI over Normalised Difference Vegetation Index because it does not become saturated as easily in high-biomass areas^61,62^. We downloaded monthly EVI data from http://reverb.echo.nasa.gov/ and calculated mean EVI for each seasonal range. We excluded all water pixels from the analyses and set all EVI values < 0.05 (indicative of non-vegetated areas) to 0.05^63^ We used dry season Landsat 8 imagery and supervised classification to generate our own fine-scale (30 m) permeant water distribution estimates for each of the clusters (full details of the procedure can be found in^64^). Based on the water distribution we generated a Euclidean distance to water layer and calculated the mean distance to water for each of the seasonal ranges (see Supplementary Information Table S2 for all extracted values).

Generalised linear mixed models^65^ (GLMM) were used to model binary data represented by ‘0’ or ‘1’. Zero represented the range elephants migrated from and ‘1’ represented the range they migrated to. The explanatory variables were; mean EVI, mean distance to water (distance to water was log transformed to normalise the distribution), and elephant density. Only six animals migrated more than once. To account for these repeated observations of migration amongst individuals, one year was randomly selected from each of these individuals. To account for repeated observations of migration within each cluster, “cluster” was added as a random effect. We formulated each set of candidate models using an all subset approach and ranked each candidate mixed model using Akaike’s information criterion (AICc)^66^. AICc was used to account for small sample size. The strength of support for the best model and alternate best models was assessed using AICc differences between the approximate best model and alternate candidate models. The Akaike weight for each candidate model also was calculated^66^.

## Electronic supplementary material

Supplementary Information
