# Supplementary material for: Partial migration in savanna elephant populations distributed across southern Africa
Source: Sci Rep. 2018 Jul 27;8:11331. doi: 10.1038/s41598-018-29724-9 (PMC6063881; doi:10.1038/s41598-018-29724-9)
Supplement: Supplementary file 1 — Supplementary Information [file 41598_2018_29724_MOESM1_ESM.docx]

**Supplementary Information**

Partial migration in savanna elephant populations distributed across southern Africa

Andrew Purdon^1, +^, Michael A. Mole^1, +^, Michael J. Chase^2^ & Rudi J. van Aarde^1, *^

^1^Affiliation, Conservation Ecology Research Unit, Department of Zoology and Entomology, University of Pretoria, Pretoria 0028, South Africa

^2^ Affiliation, Elephants Without Borders, Kasane, Botswana

* rjvaarde@zoology.up.ac.za

^+^these authors contributed equally to this work

**This file includes the following seven documents:**

1. Table S1: Summary of information relating to each classified migration.
2. Table S2: Table representing mean Enhanced Vegetation Index (EVI), mean distance from water (m) and mean number of elephants per km^2^.
3. Table S3: Tables representing the results of GLMM selection process and significance of parameters in explaining the selection of wet season ranges by migratory elephants.
4. Figure S1: Flow diagram of the methods.
5. Figure S2: Graphical example of an individual year classified as migratory using the Net squared displacement method and overlap method.
6. Figure S3: Modelled parameters from the migratory NSD model
7. Supplementary Information Materials and Methods
8. Supplementary information References

**Table S1:** Summary of information relating to each classified migration. Sex is represented by F = female or M = male); years tracked refers to the number of years of location data for each individual that was analysed in the dataset; migratory event corresponds to the ID’s 1-31 given to each migration classified and illustrated within the figures in the main text; year is the year corresponding to the starting day of the year of location data (i.e. Chobe cluster start dates were 01 Nov of every year); Leave and return days are represented by Julian days of a year.

| Elephant ID | | Sex | Years tracked | Migratory  ID | Year | Cluster | Countries migration was in | Leave day | Return day | Duration  (days) | Distance (km) | Beyond primary PA’s | Beyond primary and secondary PA’s |
| --- | --- | --- | --- | --- | --- | --- | --- | --- | --- | --- | --- | --- | --- |
| 1 | | F | 1 | 1 | 2003 | Chobe | Botswana | 331 | 206 | 240 | 73 | Yes | no |
| 2 | | M | 2 | 2 | 2004 | Chobe | Botswana | 314 | 60 | 111 | 82 | yes | no |
| 3 | | F | 1 | 3 | 2005 | Chobe | Botswana | 316 | 55 | 104 | 176 | yes | yes |
| 4 | | M | 2 | 4 | 2009 | Chobe | Botswana | 333 | 47 | 79 | 142 | yes | no |
|  | |  |  | 5 | 2010 | Chobe | Botswana | 346 | 57 | 76 | 138 | yes | no |
| 5 | | F | 2 | 6 | 2012 | Chobe | Botswana | 338 | 91 | 118 | 185 | yes | no |
|  | |  |  | 7 | 2013 | Chobe | Botswana | 347 | 215 | 233 | 165 | yes | no |
| 6 | | F | 3 | 8 | 2003 | Chobe | Botswana | 339 | 163 | 189 | 44 | yes | yes |
| 7 | | F | 2 | 9 | 2004 | Chobe | Namibia | 19 | 161 | 142 | 60 | no | no |
|  | |  |  | 10 | 2005 | Chobe | Namibia | 3 | 136 | 133 | 85 | yes | no |
| 8 | | M | 3 | 11 | 2007 | Chobe | Namibia | 42 | 200 | 158 | 54 | yes | no |
| 9 | | M | 1 | 12 | 2004 | Chobe | Namibia/Botswana | 152 | 255 | 103 | 123 | yes | yes |
| 10 | | F | 1 | 13 | 2004 | Chobe | Zimbabwe/Botswana | 343 | 180 | 202 | 90 | yes | no |
| 11 | | M | 1 | 14 | 2003 | Chobe | Namibia/Angola | 365 | 78 | 78 | 140 | yes | no |
| 12 | | M | 1 | 15 | 2006 | Chobe | Zambia/Namibia/Botswana | 46 | 226 | 180 | 169 | yes | yes |
| 13 | | F | 2 | 16 | 2012 | Chobe | Botswana/Zimbabwe | 2 | 192 | 189 | 20 | yes | no |
| 14 | | F | 3 | 17 | 2006 | Chobe | Namibia/Angola | 17 | 116 | 100 | 137 | yes | no |
| 15 | | F | 3 | 18 | 2006 | Chobe | Namibia/Zambia | 354 | 106 | 117 | 79 | yes | no |
|  | |  |  | 19 | 2007 | Chobe | Namibia/Zambia | 27 | 90 | 62 | 31 | yes | no |
| 16 | | M | 1 | 20 | 2005 | Etosha | Namibia | 113 | 172 | 59 | 109 | yes | yes |
| 17 | | F | 1 | *21 | 2002 | Etosha | Namibia | 327 | 94 | 132 | 65 | no | no |
| 18 | | F | 1 | 22 | 2002 | Etosha | Namibia | 8 | 300 | 292 | 249 | no | no |
| 19 | | F | 3 | 23 | 2014 | Limpopo | South Africa | 201 | 253 | 51 | 55 | yes | no |
| 20 | | F | 3 | 24 | 2014 | Limpopo | South Africa | 13 | 44 | 31 | 69 | no | no |
| 21 | | F | 3 | 25 | 2014 | Limpopo | South Africa | 119 | 178 | 59 | 41 | no | no |
| 22 | | F | 2 | 26 | 2013 | Limpopo | South Africa/Mozambique | 332 | 139 | 172 | 43 | yes | no |
| 23 | | F | 4 | 27 | 2004 | Luangwa | Zambia | 342 | 45 | 67 | 117 | yes | no |
|  | |  |  | 28 | 2006 | Luangwa | Zambia | 71 | 262 | 191 | 56 | yes | no |
| 24 | | M | 2 | 29 | 2008 | Niassa | Mozambique | 337 | 70 | 98 | 40 | yes | yes |
|  | |  |  | 30 | 2009 | Niassa | Mozambique | 361 | 163 | 167 | 32 | yes | yes |
| 25 | | F | 1 | *31 | 2005 | Zambezi | Zambia/Zimbabwe | 329 | 102 | 138 | 80 | yes | no |
|  |  |  |  |  |  |  |  |  |  |  |  |  |  |

**Table S2.** Table representing mean Enhanced Vegetation Index (EVI), mean distance from water (m) and mean number of elephants per km^2^ derived from count data for each seasonal range (away and start). The away seasonal range is the range the elephant migrated to (for example, seasonal range 2 in Figure S2 above). The start seasonal range is the range the elephant migrated from and returned to (for example, seasonal range 1 and 3 in Figure S2 above). The migration ID’s correspond to those in Table S2 below. If count data was unavailable for a specific area, then density was designated with an NA value.

| Migration  ID | Migration year | Seasonal range | Mean EVI | Mean distance  from water (m) | Elephant  density  (Ind/km^2^) | Survey year | Survey location | Cluster | Reference for density values |
| --- | --- | --- | --- | --- | --- | --- | --- | --- | --- |
| 1 | 2003 | away | 0.248 | 90066 | 0.109 | 2004 | Nxai Pan | Chobe | BDWNP |
| 1 | 2003 | start | 0.282 | 16506 | 0.037 | 2004 | NG/41 35D | Chobe | BDWNP |
| 2 | 2004 | away | 0.253 | 33940 | 0.047 | 2004 | NG/43 36D | Chobe | BDWNP |
| 2 | 2004 | start | 0.235 | 2773 | 0.543 | 2004 | Moremi | Chobe | BDWNP |
| 3 | 2005 | away | 0.250 | 20066 | 0.037 | 2004 | CH/11 35D | Chobe | BDWNP |
| 3 | 2005 | start | 0.278 | 9525 | 3.196 | 2004 | Chobe | Chobe | BDWNP |
| 4 | 2009 | away | 0.235 | 4564 | 0.004 | 2004 | NG/30 25D | Chobe | BDWNP |
| 4 | 2009 | start | 0.248 | 34072 | 0.092 | 2004 | Nxai Pan/Border Makgadikgadi | Chobe | BDWNP |
| 5 | 2010 | away | 0.311 | 14851 | 0.047 | 2004 | NG/34 36D | Chobe | BDWNP |
| 5 | 2010 | start | 0.285 | 31103 | 0.092 | 2004 | Makgadikgadi | Chobe | BDWNP |
| 6 | 2012 | away | 0.221 | 47007 | 0.193 | 2011 | CT/2 38 | Chobe | BDWNP |
| 6 | 2012 | start | 0.226 | 8816 | 1.997 | 2011 | Chobe | Chobe | BDWNP |
| 7 | 2013 | away | 0.244 | 50503 | 0.193 | 2011 | CT/2 38 | Chobe | BDWNP |
| 7 | 2013 | start | 0.214 | 10042 | 1.997 | 2011 | Chobe | Chobe | BDWNP |
| 8 | 2003 | away | 0.280 | 35589 | 3.473 | 2011 | North of Moremi 29 | Chobe | BDWNP |
| 8 | 2003 | start | 0.314 | 23700 | 3.473 | 2011 | North of Moremi 29 | Chobe | BDWNP |
| 9 | 2004 | away | 0.210 | 6553 | 0.089 | 2004 | Khaudum | Chobe | BDWNP |
| 9 | 2004 | start | 0.220 | 8582 | 0.089 | 2004 | Khaudum | Chobe | BDWNP |
| 10 | 2005 | away | 0.329 | 11854 | 0.089 | 2004 | Khaudum | Chobe | BDWNP |
| 10 | 2005 | start | 0.356 | 7930 | 0.089 | 2004 | Khaudum | Chobe | BDWNP |
| 11 | 2007 | away | 0.259 | 4384 | 0.014 | 2004 | Mudumu | Chobe | BDWNP |
| 11 | 2007 | start | 0.253 | 3530 | 0.014 | 2004 | Mudumu | Chobe | BDWNP |
| 12 | 2004 | away | 0.188 | 4349 | 0.014 | 2004 | Caprivi Strip (East Caprivi) | Chobe | BDWNP |
| 12 | 2004 | start | 0.149 | 10683 | 3.196 | 2004 | Chobe | Chobe | BDWNP |
| 13 | 2004 | away | 0.225 | 23627 | 0.543 | 2007 | Hwange 33D | Chobe | BDWNP |
| 13 | 2004 | start | 0.267 | 8741 | 2.255 | 2007 | Hwange | Chobe | BDWNP |
| 14 | 2003 | away | 0.315 | 44717 | 0.398 | 2004 | Bwabwata | Chobe | BDWNP |
| 14 | 2003 | start | 0.303 | 36204 | 0.398 | 2004 | Bwabwata | Chobe | BDWNP |
| 15 | 2006 | away | 0.244 | 6816 | 4.367 | 2005 | North of Chobe 31D | Chobe | BDWNP |
| 15 | 2006 | start | 0.262 | 21191 | 0.088 | 2005 | Sioma Ngwezi | Chobe | BDWNP |
| 16 | 2012 | away | 0.243 | 5565 | 8.517 | 2005 | Kasane FR 32 | Chobe | BDWNP |
| 16 | 2012 | start | 0.234 | 9294 | 8.517 | 2005 | Kasane FR 32 | Chobe | BDWNP |
| 17 | 2006 | away | 0.242 | 12176 | 0.188 | 2005 | Luiana | Chobe | BDWNP |
| 17 | 2006 | start | 0.270 | 17354 | 0.188 | 2005 | Luiana | Chobe | BDWNP |
| 18 | 2006 | away | 0.341 | 7292 | 0.088 | 2004 | Sioma Ngwezi | Chobe | BDWNP |
| 18 | 2006 | start | 0.311 | 5194 | 0.014 | 2004 | Mudumu | Chobe | BDWNP |
| 19 | 2007 | away | 0.359 | 14339 | 0.014 | 2004 | Caprivi | Chobe | BDWNP |
| 19 | 2007 | start | 0.348 | 10917 | 0.014 | 2004 | Lubuta/Mashi | Chobe | BDWNP |
| 20 | 2005 | away | 0.190 | 34546 | NA |  |  | Etosha |  |
| 20 | 2005 | start | 0.196 | 52011 | NA |  |  | Etosha |  |
| 21 | 2002 | away | 0.189 | 12279 | 0.093 | 2002 | Etosha NP | Etosha | CERU database (van Aarde et al.) |
| 21 | 2002 | start | 0.172 | 8082 | 0.093 | 2002 | Etosha NP | Etosha | CERU database (van Aarde et al.) |
| 22 | 2002 | away | 0.162 | 20519 | 0.093 | 2002 | Etosha NP | Etosha | CERU database (van Aarde et al.) |
| 22 | 2002 | start | 0.160 | 20287 | 0.093 | 2002 | Etosha NP | Etosha | CERU database (van Aarde et al.) |
| 23 | 2014 | away | 0.256 | 3663 | 0.899 | 2015 | Kruger National Park | Limpopo | S. Ferreira, personal communication. |
| 23 | 2014 | start | 0.238 | 2616 | NA |  |  | Limpopo |  |
| 24 | 2014 | away | 0.265 | 3821 | 0.899 | 2015 | Kruger National Park | Limpopo | S. Ferreira, personal communication. |
| 24 | 2014 | start | 0.348 | 2482 | 0.899 | 2015 | Kruger National Park | Limpopo | S. Ferreira, personal communication. |
| 25 | 2014 | away | 0.237 | 3350 | 0.899 | 2015 | Kruger National Park | Limpopo | S. Ferreira, personal communication. |
| 25 | 2014 | start | 0.232 | 4336 | 0.899 | 2015 | Kruger National Park | Limpopo | S. Ferreira, personal communication. |
| 26 | 2013 | away | 0.316 | 6282 | 0.101 | 2010 | Limpopo National Park | Limpopo | S. Ferreira, personal communication. |
| 26 | 2013 | start | 0.304 | 3222 | 0.899 | 2015 | Kruger National Park | Limpopo | S. Ferreira, personal communication. |
| 27 | 2004 | away | 0.498 | 7002 | 0.418 | 1999 | Munyamadzi | Luangwa | Blanc et al. |
| 27 | 2004 | start | 0.494 | 6575 | 0.518 | 2002 | South Luangwa NP | Luangwa | Blanc et al. |
| 28 | 2006 | away | 0.285 | 3862 | 0.518 | 2002 | South Luangwa NP | Luangwa | Blanc et al. |
| 28 | 2006 | start | 0.288 | 4412 | 0.418 | 1999 | Munyamadzi | Luangwa | Blanc et al. |
| 29 | 2008 | away | 0.466 | 13110 | 0.084 | 2004 | Quirimbas | Niassa | Dunham, K.M. |
| 29 | 2008 | start | 0.482 | 5751 | 0.084 | 2004 | Quirimbas | Niassa | Dunham, K.M. |
| 30 | 2009 | away | 0.407 | 16946 | 0.084 | 2004 | Quirimbas | Niassa | Dunham, K.M. |
| 30 | 2009 | start | 0.401 | 4791 | 0.084 | 2004 | Quirimbas | Niassa | Dunham, K.M. |
| 31 | 2005 | away | 0.442 | 4459 | 1.549 | 2003 | Hurungwe | Zambezi | historical data cited in GEC report |
| 31 | 2005 | start | 0.444 | 3736 | 1.959 | 2003 | Mana Pools | Zambezi | historical data cited in GEC report |

**Table S3.**

Table S3a. Summary of AICc selection approach for candidate GLMM used to test whether the selection of wet season ranges could be explained by differences in EVI, distance to water or elephant density

| model | df | logLik | AICc | AICc Difference | AICc weight |
| --- | --- | --- | --- | --- | --- |
| (Null) | 2 | -34.66 | 73.57 | 0 | 0.32 |
| density | 3 | -34.01 | 74.54 | 0.97 | 0.2 |
| water | 3 | -34.27 | 75.07 | 1.5 | 0.15 |
| EVI | 3 | -34.65 | 75.81 | 2.24 | 0.11 |
| density + water | 4 | -33.69 | 76.27 | 2.7 | 0.08 |
| density + EVI | 4 | -33.98 | 76.84 | 3.27 | 0.06 |
| EVI + water | 4 | -34.27 | 77.43 | 3.86 | 0.05 |
| denisty + EVI + water | 5 | -33.69 | 78.74 | 5.17 | 0.02 |

Table S3b. Summary of the GLMM model-averaged coefficients for each parameter in explaining the selection of wet season range by migratory elephants

|  | Estimate | Std. Error | Adjusted SE | z value | Pr(>\|z\|) |
| --- | --- | --- | --- | --- | --- |
| (Intercept) | -0.50605 | 2.13923 | 2.17859 | 0.232 | 0.816 |
| Elephant density | -0.01391 | 0.02738 | 0.02779 | 0.5 | 0.617 |
| Distance from permanent water | 0.08118 | 0.21283 | 0.21668 | 0.375 | 0.708 |
| EVI | -0.11541 | 1.76755 | 1.81388 | 0.064 | 0.949 |

**
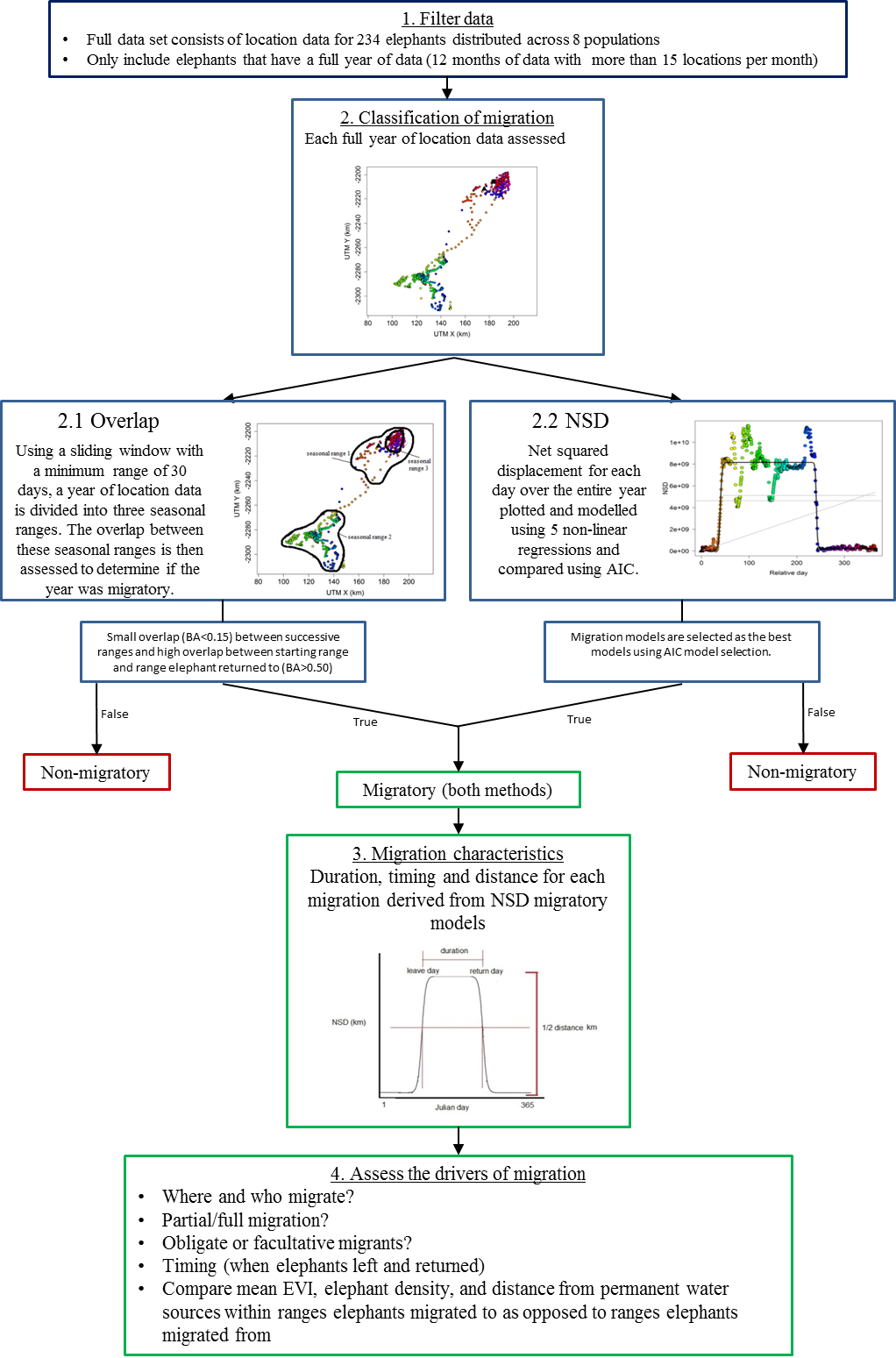
Figure S1:** Flow diagram of the methodological framework outlining the general methods and analytical routines undertaken in filtering elephant location data, classifying elephant migration and assessing patterns and drivers of elephant migration. For more detailed examples of the classification methods as well as the extraction of migration characteristics, please see Figure S2 and Figure S3 in this supplementary document.

**Figure S2:** Graphical example of an individual year classified as migratory using the Net squared displacement (NSD; Bunnefeld *et al.* 2011) method and overlap method (Cagnacci *et al.* 2016). The figures are for illustrative purposes. Fig. S2a illustrates NSD (m) over the course of a year. Fig. S2b represents the same individual’s locational data for the same year and the corresponding seasonal ranges (in this case for illustration purposes, 95% utilisation distributions) assigned using the shifting window of the overlap method. The coloured dots are a corresponding time series between the two plots. This individual represents individual 10 and migratory ID 13 in Fig. 3 and Fig. 5 of the main document, and Supplementary file 1 Table B in this document.

NSD measures the cumulative squared displacement from a starting location (Bunnefeld *et al.* 2010). The below example (Fig. S2a) was best fitted by the migration non-linear model. The overlap of seasonal ranges delimits seasons by shifting time windows (resolution of one month) and then computes the degree of overlap between the seasons using the Bhattacharyya’s affinity index (BA) index. The example below shows 3 seasonal ranges. This individual was classified as migratory as successive seasonal ranges 1 and 2 had a BA index < 0.15 as well as a BA index > 0.5 between seasonal range 1 and the return seasonal range (seasonal range 3).


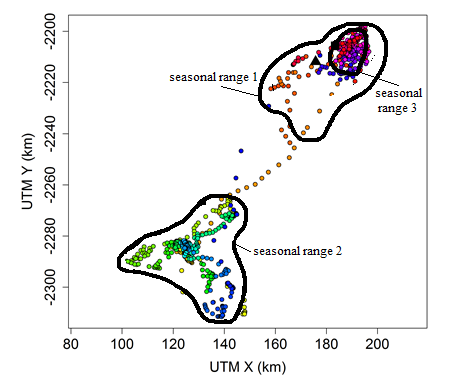

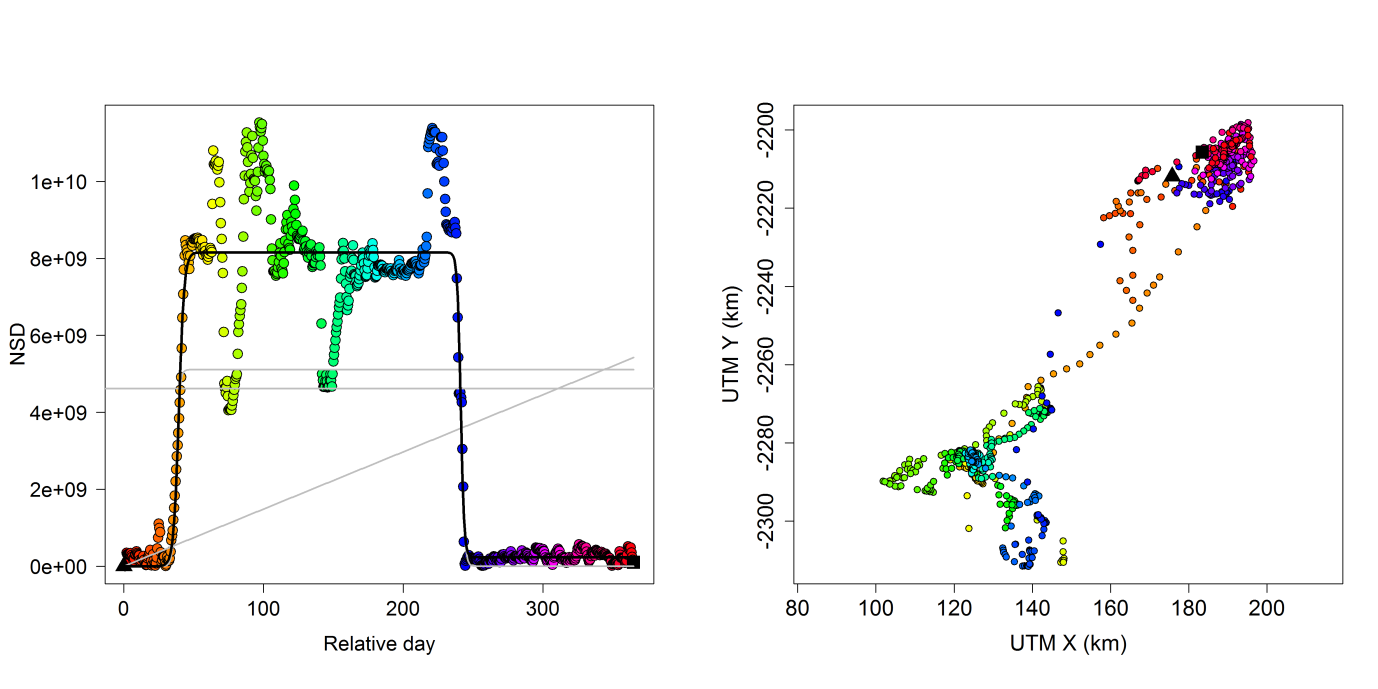


a)

b)

**Figure S3:** For all years classified as migratory in both the NSD and overlap methods, we assessed the patterns of migration using the modelled parameters from the migratory NSD model. Patterns of the migrations that we extracted were duration, timing and distance. The one-way migration distance was calculated as the asymptotic height of the modelled NSD and the leaving and return date were calculated as the day at which the migration reached half its asymptotic height.


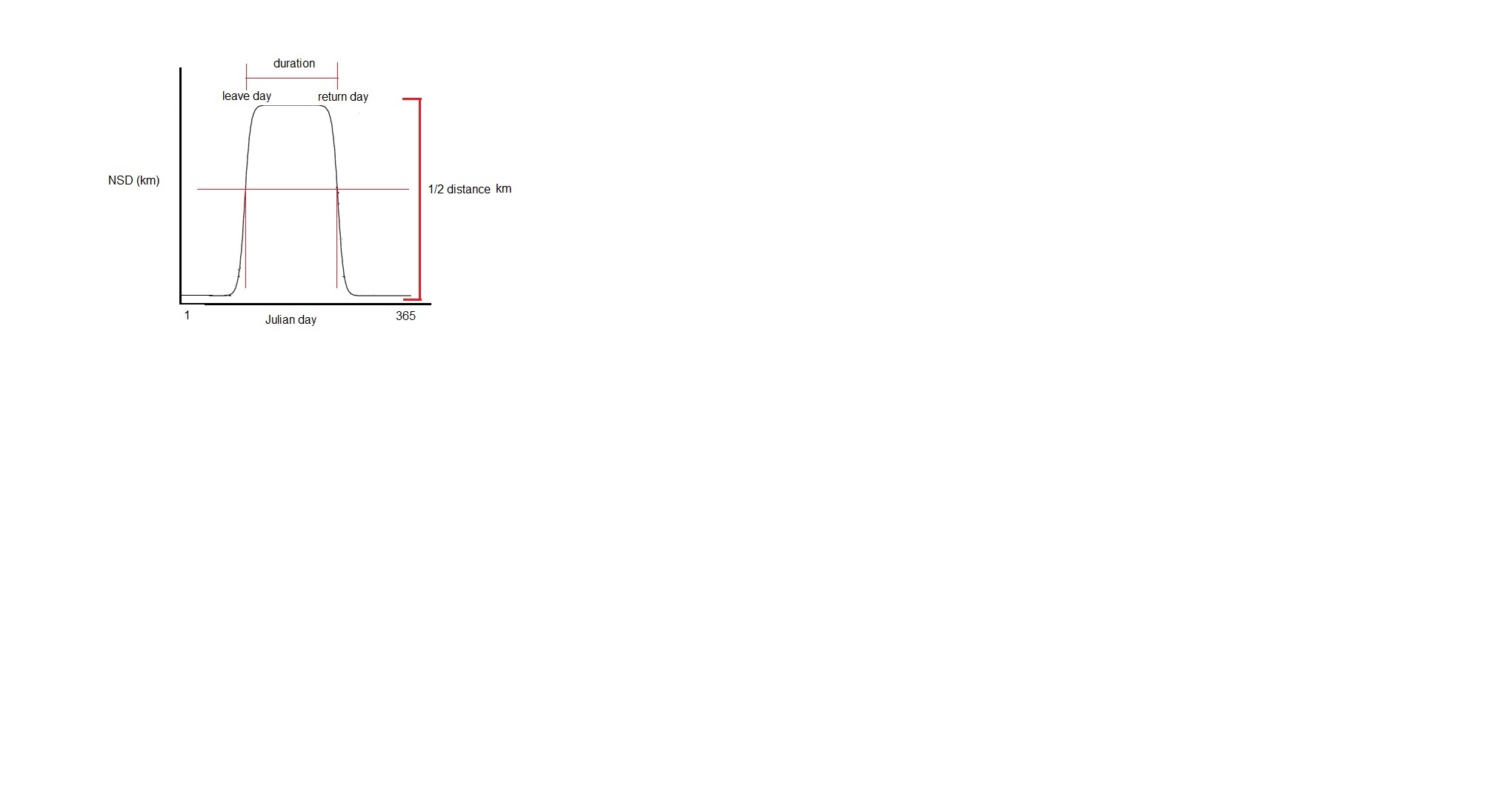


**Supplementary Information Materials and Methods**

**Environmental variables for Figure 4**

We downloaded monthly EVI data from http://reverb.echo.nasa.gov/ and calculated long-term mean monthly EVI (2000-2015) for the study area (Figure 1). The long-term mean was most appropriate to capture broad-scale differences in EVI between months. We excluded all water pixels from the analyses and set all EVI values < -.05 (indicative of non-vegetated areas) to -.05 (Teitelbaum *et al.* 2015). Similarly, for rainfall, we downloaded monthly rainfall layers from http://chrsdata.eng.uci.edu/ (Ashouri *et al.* 2015) and calculated long-term mean monthly (2000-2015) rainfall for the study area.

**Supplementary Information References**

1. Ashouri, H et al. PERSIANN-CDR: Daily precipitation climate data record from multisatellite observations for hydrological and climate studies. *Bull. Am. Meteorol. Soc.* **96(1),** 69-83 (2015).
2. Bunnefeld, N. et al. A model‐driven approach to quantify migration patterns: individual, regional and yearly differences. *J. Anim. Ecol*. **80(2),** 466-476 (2011).
3. Cagnacci, F. et al. How many routes lead to migration? Comparison of methods to assess and characterize migratory movements. *J. Anim. Ecol.* **85(1),** 54-68 (2016).
4. Duffy, J. P. & Pettorelli, N. Exploring the relationship between NDVI and African elephant population density in protected areas*. Af.r J. Ecol*. **50 (4),** 455–463 (2012).
5. Huete, A et al. Overview of the radiometric and biophysical performance of the MODIS vegetation indices. *Remote Sens. Environ.* **83(1),** 195-213 (2002).
6. Pettorelli, N. et al. Using the satellite-derived NDVI to assess ecological responses to environmental change. *Trends Ecol. Evolut.* **20(9),** 503-510 (2005).
7. Teitelbaum, C. S. et al. How far to go? Determinants of migration distance in land mammals. *Ecol. Lett*. **18(6),** 545-552 (2015).
